# Visible-light driven Photoelectrochemical Immunosensor Based on SnS_2_@mpg-C_3_N_4_ for Detection of Prostate Specific Antigen

**DOI:** 10.1038/s41598-017-04924-x

**Published:** 2017-07-05

**Authors:** Yifeng Zhang, Yixin Liu, Rongxia Li, Malik Saddam Khan, Picheng Gao, Yong Zhang, Qin Wei

**Affiliations:** 1 Key Laboratory of Interfacial Reaction & Sensing Analysis in Universities of Shandong, University of Jinan, Jinan, 250022 P.R. China; 2 Shandong Liyuan Kangsai Environmental Consulting Co. Ltd., Shandong, P.R. China

## Abstract

Herein, a novel label-free photoelectrochemical (PEC) immunosensor based on SnS_2_@mpg-C_3_N_4_ nanocomposite is fabricated for the detection of prostate specific antigen (PSA) in human serum. Firstly, mesoporous graphite-like carbon nitride (mpg-C_3_N_4_) with carboxyl groups is synthesized successfully which possesses high specific surface area and large pore volume. Then, SnS_2_ as a typical n-type semiconductor with weak photoelectric conversion capability is successfully loaded on carboxylated mpg-C_3_N_4_ to form a well-matched overlapping band-structure. The as-synthesized SnS_2_@mpg-C_3_N_4_ nanocomposite performs outstanding photocurrent response under visible-light irradiation due to low recombination rate of photoexcited electron-hole pairs, which is transcend than pure SnS_2_ or pure mpg-C_3_N_4_. It is worth noting that SnS_2_@mpg-C_3_N_4_ nanocomposite is firstly employed as the photoactive material in PEC immunosensor area. The concentration of PSA can be analyzed by the decrease in photocurrent resulted from increased steric hindrance of the immunocomplex. Under the optimal conditions, the developed PEC immunosensor displays a liner photocurrent response in the range of 50 fg·mL^−1^ ~ 10 ng·mL^−1^ with a low detection limit of 21 fg·mL^−1^. Furthermore, the fabricated immunosensor with satisfactory stability, reproducibility and selectivity provides a novel method for PSA determination in real sample analysis.

## Introduction

Prostate specific antigen (PSA), a kind of kallikrein-like serine protease, is secreted by prostatic epithelial cells. Due to the abnormal level of PSA can suggest a problem in the prostate, such as prostatitis, benign hyperplasia, even prostate cancer^1^, sensitive detection of PSA in human serum has been involved a large interest in the field of analytical chemistry since it was first described by Hara in 1971^2^. Many methods have been developed for the detection of PSA, such as Fluorescence Immunoassay^3^, electrochemiluminescence (ECL)^4^, surface acoustic wave^5^ and so on. Although high sensitivity has been achieved, these methods are laborious, expensive, time-consuming. Among kinds of newly developed analytical techniques, the photoelectrochemical (PEC) immunosensor have numerous advantages, such as good selectivity, high sensitivity, fast analysis speed and low cost, *etc* ^6–8^. Furthermore, benefitting from the separation and the different energy forms of the excitation source and detection signal, PEC analysis has higher selectivity and sensitivity than the conventional electrochemical analytical method due to the reduced background signals^9, 10^. Thus, the enhanced detection signals, getting from better PEC efficiency, can lower detection limit and increase the sensitivity of the PEC sensor^11, 12^.

To get high PEC efficiency, it is necessary to develop a photoactive material with high photoelectric transformation efficiency. SnS_2_, which has a CdI_2_-related crystal structure, is a typical n-type semiconductor with a band gap about 2.44 eV^13, 14^. SnS_2_ can remain inert in the non-alkaline solution and have certain oxidative and thermal stability in air and these merits promise it a very wide range of applications, such as phototransistors^15, 16^, lithium-ion batteries^17, 18^, dye-sensitized solar cells^19, 20^, photocatalysts^21, 22^ and so on. However, the low photoelectric conversion efficiency limits its further practical applications due to the high recombination rate of photoexcited electron-hole pairs. To improve this situation for better applications, many methods have been carried out, such as doping with other elements, morphology control and compositing with other semiconducting materials^23–25^. Among them, compositing with other semiconductor materials is one of the most common and effective methods. Nevertheless, most of the semiconductors used for compositing with the others are either environment unfriendly or costly. For example, the synthetize of CdS and CdSe always involves toxic reagent, and the application of TiO_2_ are costly^26, 27^.

Graphite-like carbon nitride (g-C_3_N_4_) with a band gap of ~2.7 eV, connected by a tertiary amine two-dimensional nanosheet structure, has attracted adequate attention due to its magnificent properties such as controllable morphology, cheapness, abundance and stability^27, 28^. Among various morphologies of g-C_3_N_4_, mesoporous g-C_3_N_4_ (mpg-C_3_N_4_) has more stable activity, large specific surface area and multiple scattering effects, which can principally enhance the material’s light-capturing ability and reactant sorption capacity^29^. As a result, mpg-C_3_N_4_ has been widely used in photocatalysis^30–33^. Benefiting from the large specific surface area and porous structure, it is much easier for sufficient *in situ* assembly of SnS_2_ on the surface of the mpg-C_3_N_4_. What’s more, the introduction of carboxyl groups makes mpg-C_3_N_4_ have a better hydrophilicity resulting in increasing dispersibility in water, which is conductive to the stability of synthetic materials. The as-synthesized SnS_2_@mpg-C_3_N_4_ nanocomposite had a better PEC response benefiting from the well-matched overlapping band potentials which greatly reduced the recombination rate of photoexcited electron-hole pairs, and as a consequence, high photocurrent response was produced. In addition, the composites have a wider absorption band in visible light which increased the possibility of practical application.

**Figure 1:**
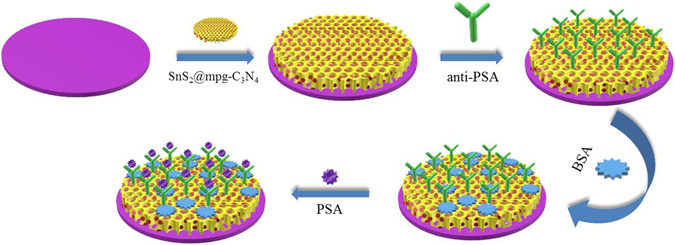
Schematic diagram of the immunosensor construction process.

In this work, a novel PEC immunosensor was fabricated based on SnS_2_@mpg-C_3_N_4_ nanocomposite for sensitive detection of PSA. As shown in the Fig. 1, mpg-C_3_N_4_, with the large specific surface area, is employed as the carrier to load SnS_2_ by hydrothermal reaction. Antibody of prostate specific antigen (anti-PSA) is immobilized onto ITO electrodes modified by the SnS_2_@mpg-C_3_N_4_ nanocomposite via the classic 1-ethyl-3-(3-dimethylaminopropyl) carbodiimide hydrochloride (EDC) coupling reactions between carboxyl groups on the surface of the SnS_2_@mpg-C_3_N_4_ nanocomposite and the amino groups of the antibody. The PSA concentration could be measured through the decrease in photocurrent intensity resulting from its immune response to the anti-PSA. What’s more, as an electron donor, ascorbic acid (AA) inhibited the recombination of photoexcited electron-hole pairs, and to some extent it increased the photocurrent.

## Results and Discussion

### Characterization of SnS_2_, mpg-C_3_N_4_, and SnS_2_@mpg-C_3_N_4_ nanocomposite

**Figure 2:**
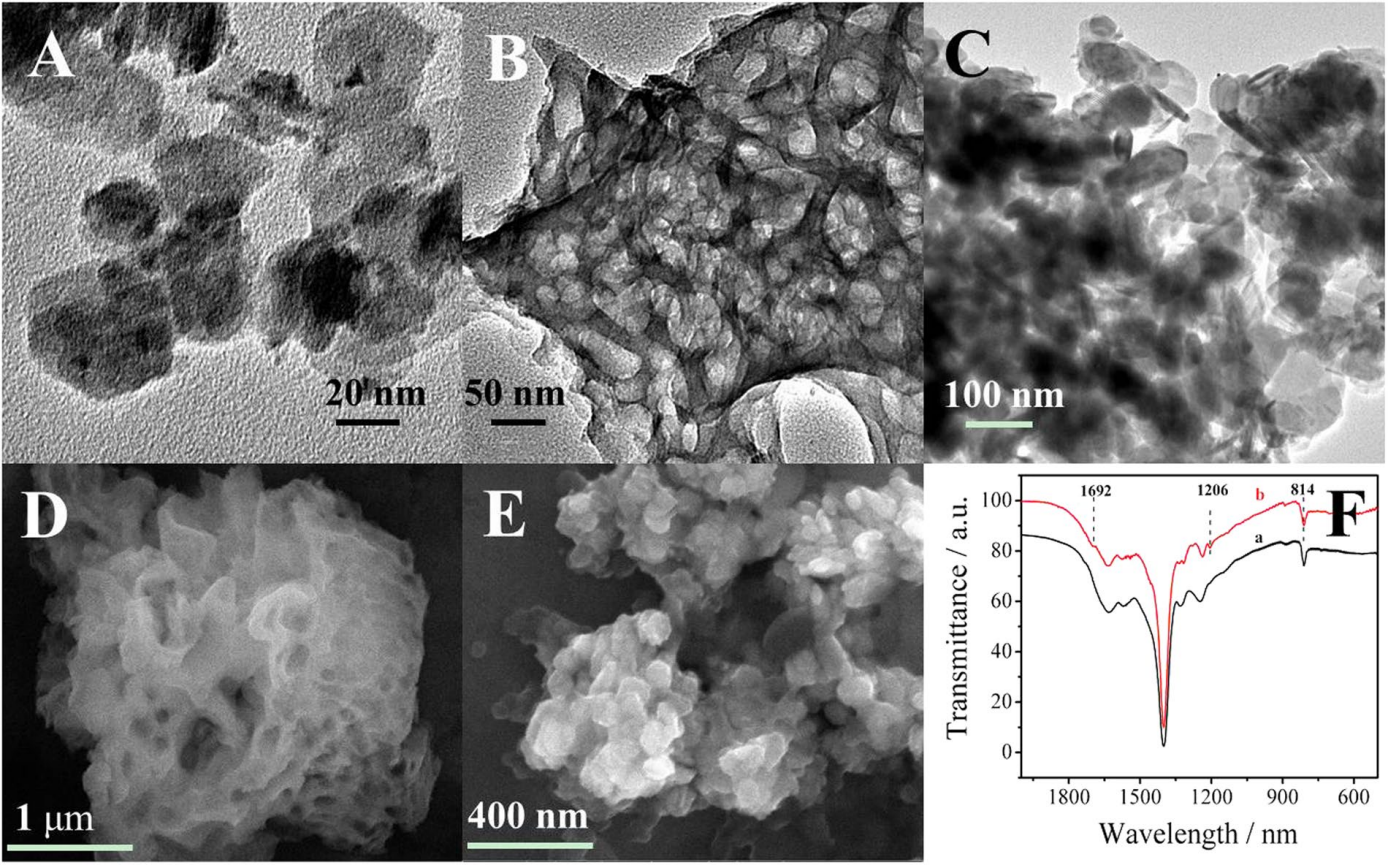
TEM image of (**A**) SnS_2_, (**B**) mpg-C_3_N_4_. (**C**) SnS_2_@ mpg-C_3_N_4_. FESEM image of (**D**)mpg-C_3_N_4_, (**E**) SnS_2_@ mpg-C_3_N_4_. (**F**) FT-IR spectrum of (a) mpg-C_3_N_4_ and (b) carboxylated mpg-C_3_N_4_.

The morphological structures of the as-synthesized materials were investigated by transmission electron microscope (TEM) and field emission scanning electron microscope microscope (FESEM). As shown in Fig. 2A, the TEM image of SnS_2_ reveals that SnS_2_ is a kind of sheet material. The morphological structures of mpg-C_3_N_4_ shown in Fig. 2B and D indicates that mpg-C_3_N_4_ has a porous structure. Comparing Fig. 2E with Fig. 2D, it is clearly seen that the flakes of SnS_2_ attaching on the surface of mpg-C_3_N_4_, which indicates SnS_2_@mpg-C_3_N_4_ nanocomposite has been synthesized successfully, and the porous structure of mpg-C_3_N_4_ can load a large amount of SnS_2_. Fourier Transform Infrared Spectoscopy (FT-IR) was used to detect the introduction of carboxyl groups of mpg-C_3_N_4_ and the spectrum. As shown in Fig. 2F (curve a), the absorption bands in the 1200 ~ 1700 cm^−1^ region are attributed to the typical stretching modes of CN of mpg-C_3_N_4_, and the absorption peak at 814 cm^−1^ is the characteristic absorption peak of the triazine units^34^. Compared with mpg-C_3_N_4_, the FT-IR spectra of carboxylated mpg-C_3_N_4_ (curve b) has a characteristic absorption peak at 1692 cm^−1^ and 1206 cm^−1^ corresponding to the stretching mode of C=O band and the stretching mode of C-OH, which prove that carboxyl groups has been introduced successfully^34^.

In addition, the crystalline structure of as-synthesized materials was detected with X-ray Diffraction (XRD). As shown in Supplementary Fig. S1A, the diffraction peaks at 15°, 28.2°, 32.1°, 49.9°, 52.5° are the characteristic peaks of hexagonal phase SnS_2_ (JCPDS No. 23–677)^35^. After compounded with mpg-C_3_N_4_, a new weak diffraction peak appears at 27.4°, which corresponds to the (002) plane of mpg-C_3_N_4_^34^. UV–vis absorption spectroscopy (Supplementary Fig. S1B) demonstrates that SnS_2_@mpg-C_3_N_4_ nanocomposite has a broader absorption range in the visible light than that of SnS_2_ or mpg-C_3_N_4_, which also proves the successful formation of the composites of the two materials. The figure of nitrogen adsorption–desorption isotherm of synthesized mpg-C_3_N_4_ is shown in supplementary Fig. S2 and the measured BET surface area is around 43.0532 m²·g^−1^.

### Characterization of the PEC immunosensor fabricating process

**Figure 3:**
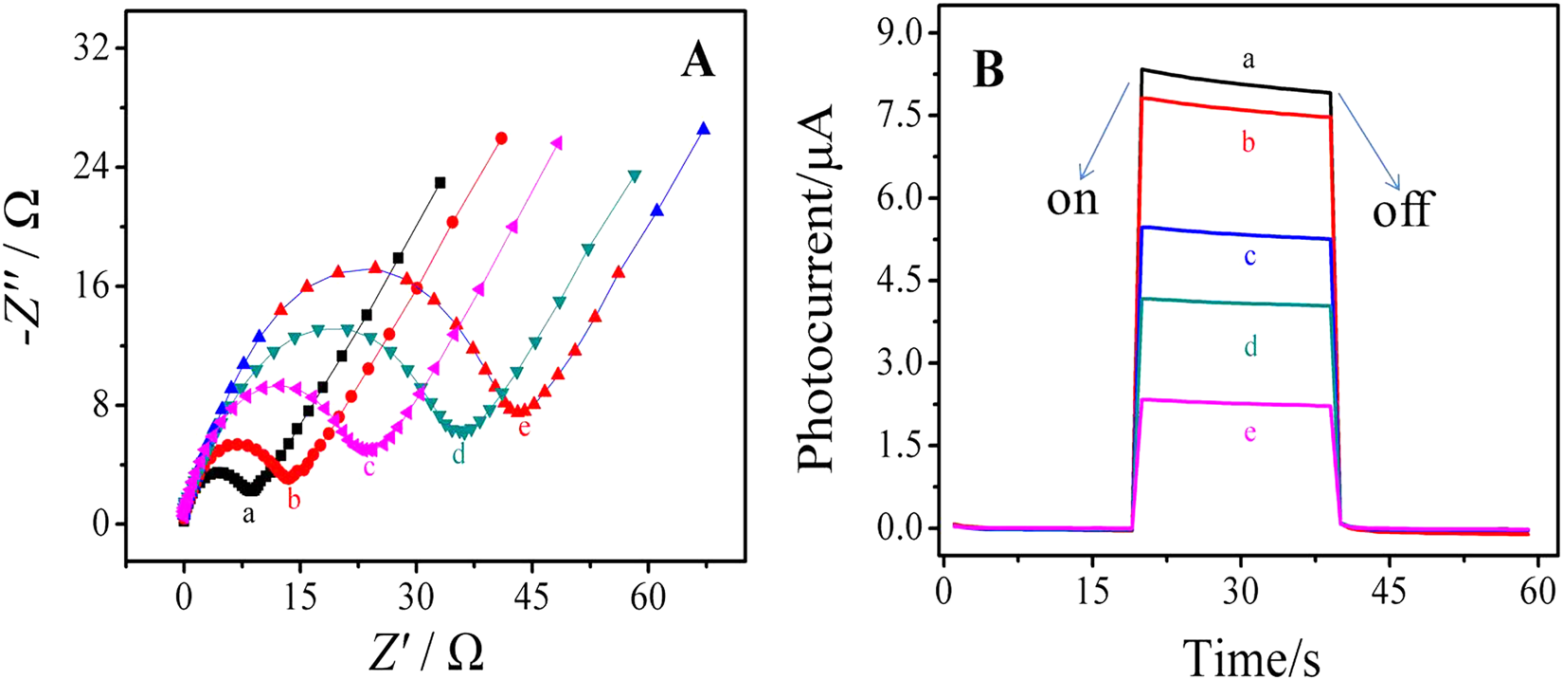
(**A**) Electrochemical impedance Nyquist plot and (**B**) corresponding photocurrent of the modified electrodes: (a) SnS_2_@mpg-C_3_N_4_, (b) SnS_2_@mpg-C_3_N_4_/(EDC/NHS), (c) SnS_2_@mpg-C_3_N_4_/(EDC/NHS)/anti-PSA, (d) SnS_2_@mpg-C_3_N_4_/(EDC/NHS)/anti-PSA/BSA, (e) SnS_2_@mpg-C_3_N_4_/(EDC/NHS)/anti-PSA/BSA/PSA.

The electrodes had different interface properties and photocurrent responses at different modification stages, which were characterized by electrochemical impedance spectroscopy (EIS) (Fig. 3A) and photocurrent measurement (Fig. 3B) respectively.

In the EIS experiments, our instrument parameters of frequency range and amplitude were set as 100 mHz ~ 100 KHz and 5 mV respectively. The bias voltage remained the default 0 V. The buffer solution used in the tests was a kind of mixed solution containing 2.5 mmol·L^−1^ K_3_[Fe(CN)_6_], 2.5 mmol·L^−1^ K_4_[Fe(CN)_6_] and 0.1 mol·L^−1^ KCl. As shown in Fig. 3A, bare electrode has the smallest diameter (curve a) of the semicircle in high frequency area, which suggests that it has good electrical conductivity. Along with the decoration of SnS_2_@mpg-C_3_N_4_ nanocomposite on the surface of ITO electrodes, the diameter of the semicircle becomes much bigger (curve b) because the electron transfer is inhibited by the semiconductor materials. After that, with anti-PSA (curve c) and Bovine serum album (BSA) (curve d) dropped successively, the diameters increase further, which is owing to the non-conductive effect of the proteins and suggests the successful modification of every step. Because of the immune complexes can greatly obstruct the transfer of the electrons to the electrode as detecting PSA, the resistance is further increased (curve e).

Figure 3B shows the PEC immunosensor fabricating process characterized by photocurrent measurement. The electrode modified with SnS_2_@mpg-C_3_N_4_ nanocomposite has significantly enhanced photocurrent response (curve a), which is attributed to the high photoelectric conversion efficiency of SnS_2_@mpg-C_3_N_4_. After the decoration of EDC/N-hydroxysuccinimide (NHS), the photocurrent response is slightly reduced (curve b), which indicats that EDC/NHS is only used for activating the carboxyl groups on the surface of the material and has little effect on the diffusion of AA^36^. Then, the photocurrent responds decrease gradually along with the anti-PSA anchoring (curve c), BSA blocking (curve d), and PSA binding process (curve e), which might be owing to the insulating effect of these proteins.

### Mechanism exploration

**Figure 4:**
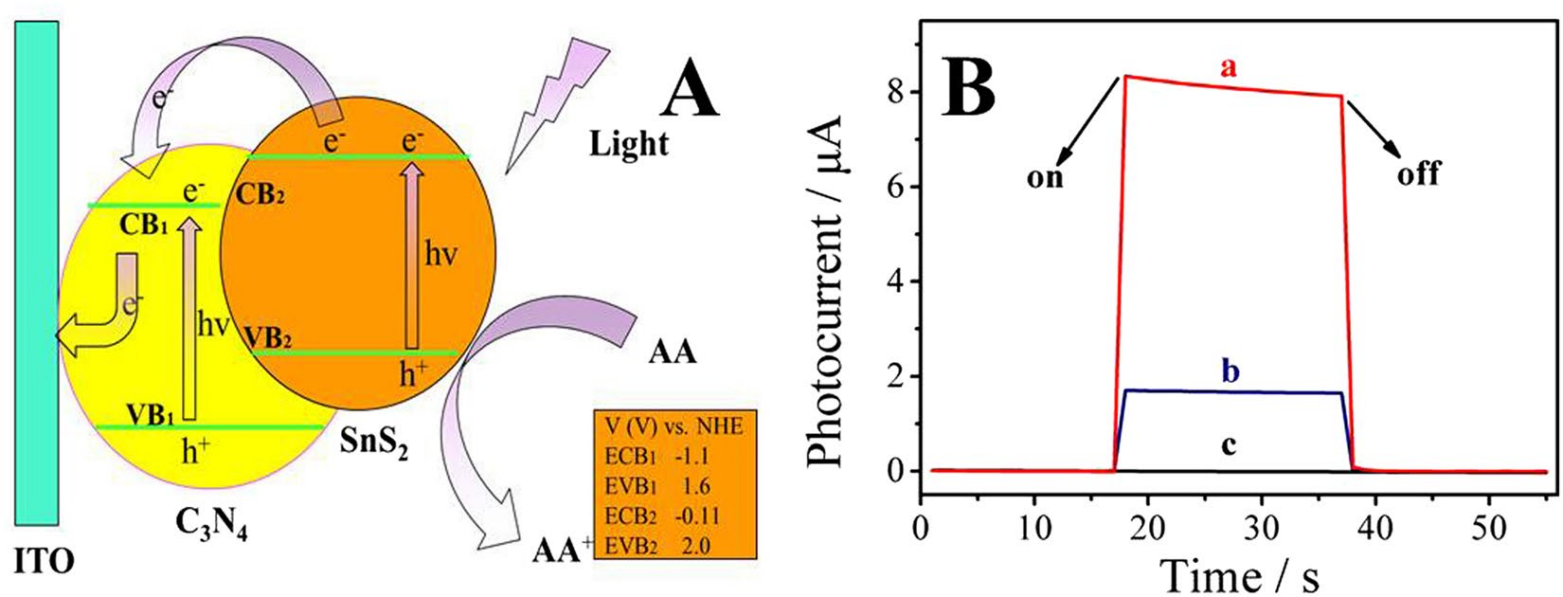
(**A**) The photocurrent generation mechanism of SnS_2_@mpg-C_3_N_4_ modified electrodes. (**B**) The photocurrent responses of (a) SnS_2_@mpg-C_3_N_4_, (b) SnS_2_ and (c), carboxylated mpg-C_3_N_4_.

As shown in Fig. 4A, after the composite of the two materials, the photoexcited electrons of SnS_2_ could quickly transfer to the conduction band of mpg-C_3_N_4_, which reduced the electron-hole pairs’ recombination of SnS_2_ and led to high separation efficiency^37–39^. The corresponding photocurrent responds of SnS_2_, mpg-C_3_N_4_ and SnS_2_@mpg-C_3_N_4_ were displayed in the Fig. 4B. It could be seen that the nanocomposite SnS_2_@mpg-C_3_N_4_ (curve a) had better photocurrent response than pure SnS_2_ (curve b) or pure carboxylated mpg-C_3_N_4_ (curve c), which proved that the photoelectric efficiency was improved.

### Effect of pH and AA concentration on the PEC immunosensor

The pH of PBS had a great influence on the sensitivity of the sensor because a large number of biologically active proteins were used in the construction of the sensor. The effect of pH on the PEC immunosensor for PSA detection was examined within the range of 5.5 ~ 8.0 in this paper and the results were showed in Supplementary Fig. S3A. It is clear that the photocurrent reaches to the maximum at pH = 7.4 and the photocurrent is reduced regardless of pH value increases or decreases. Thus, the most appropriate pH value for PSA detection is 7.4. As an electron donor, AA played a decisive role to the photocurrent response, which suggested that it was necessary to study the effect of AA concentration on the PEC immunosensor. Supplementary Fig. S3B shows the effect of different concentration of AA on the immunosensor. It can be seen that the photocurrent in 0.02 ~ 0.2 mol·L^−1^ has been increasing and begins to decrease with increasing concentration after reaching the highest at 0.2 mol·L^−1^. Thus, 0.2 mol·L^−1^ was the best concentration of AA for the detection of PSA. The reason for this trend was that AA could not provide enough electrons to combine with photoexcited holes to reduce the recombination rate of photoexcited electron-hole pairs when the concentration of AA was low, and when the concentration was high, the transmittance of the solution were deskilled, which decreased light intensity^40^.

### PEC detection of PSA

**Figure 5:**
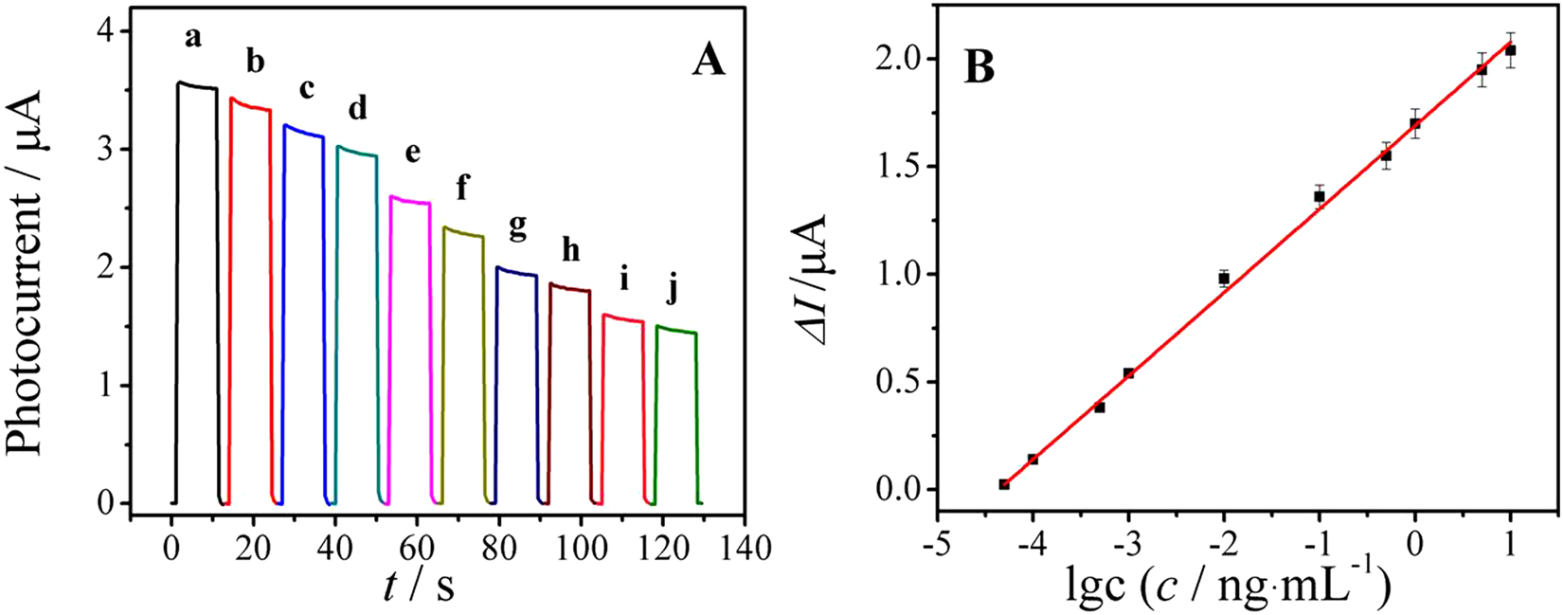
(**A**) Photocurrent responses of the PEC immunosensor with different concentrations of PSA: (a to j) 0.05, 0.1, 0.5, 1, 10, 100, 500, 1000, 5000, 10000 pg·mL^−1^. (**B**) The logarithmic calibration curve for PEC immunosensor for the detection of different concentrations of PSA.

Under the optimal conditions, different concentrations of PSA solutions were detected and showed a certain relation with photocurrent in the range of 50 fg·mL^−1^~10 ng·mL^−1^. As shown in Fig. 5B, the logarithm of PSA concentration and the decrease of the photocurrent shows a good linear relationship, and the equation is ∆*I* = 0.3875 lg*c* + 1.6907 with a correlation coefficient of 0.997. The limit of the detection was experimentally found to be 21 fg·mL^−1^ (S/N = 3). What’s more, a comparison with other methods in PSA detection was made in this paper and the statistics were shown in Supplementary Table S1. Comparing with other methods, we can see that the as-fabricated PEC immunosensor for PSA has a lower detection limit.

### Stability, reproducibility and selectivity of the sensor

**Figure 6:**
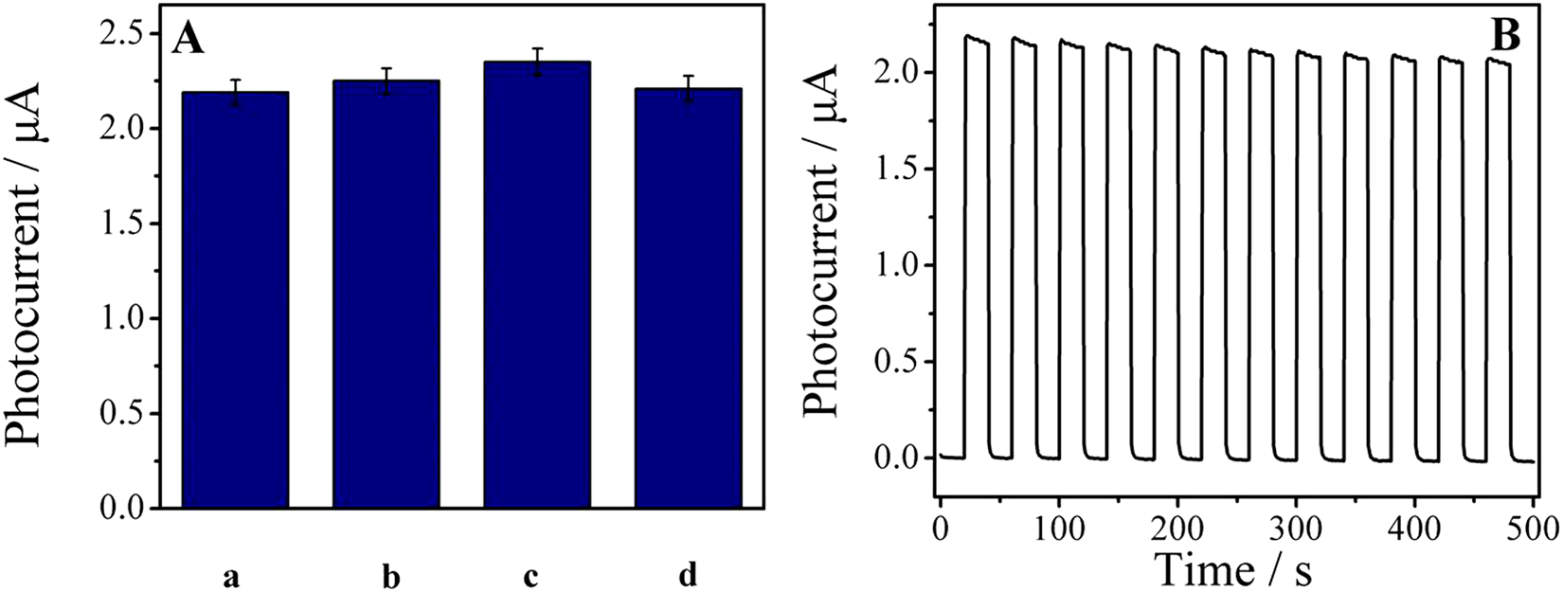
(**A**) The selectivity of the sensor (a) PSA, (b) PSA + CEA, (c)PSA + lgG, and (d) PSA + BSA. (**B**) The stability of the sensor.

There were many indicators of the practical application of the sensor. The stability, reproducibility and selectivity of the sensor were tested in this work. In order to test the selectivity of the immunesensor, some representative antigens were selected as the interferents including carcinoembryonic antigen (CEA), human immunoglobulin antigen (lgG) and BSA. Sample solution containing 100 pg·mL^−1^ PSA solution and 10 ng·mL^−1^ interferent was used to investigate the sensing performance test, and the results were shown in Fig. 6A. It can be seen that the interferent only display a small change, which suggests that the sensor has acceptable selectivity in the detection of PSA.

Inter-assay and intra-assay relative standard deviation (RSD) are important signs of reproducibility. Analysis of experimental results showed that the RSDs of the inter-assay of the five electrodes prepared with the same sample under the same conditions were 3.8%, 5.1%, and 4.7% respectively, and when the concentration of PSA was 10, 100 and 1000 pg·mL^−1^, the RSDs were 3.6%, 4.5%,and 3.9%. All these results indicated the satisfactory reproducibility for PSA detection. The stability of the sensor was measured by recording the change of photocurrent when the light source was switched on and off for several times in the case of a PSA concentration of 100 pg·mL^−1^, and the result was shown in Fig. 6B. A conclusion can be drawn from the figure that the sensor almost generated the same photocurrent value each time when the light was on, and the RSD of the photocurrent was only 1%, which indicated good stability of the sensor.

### Real sample analysis

In order to investigate the feasibility of the sensor, the content of PSA of the human serum samples were used to test and perform standard addition experiments. We tested the concentration of PSA in serum that was diluted several times at first and then it were mixed with the standard PSA solution of different concentrations to do the test (Table 1). It can be seen from the table that the average recovery rates of the sensor were 98.4%~102.5%, and the relative standard deviation is 2.5%~3.6%. Experimental results showed that the proposed PEC immunosensor had a promising application in clinical analysis of PSA.

**Table 1:** Results for the detection of PSA in serum samples.

Serum samples content (pg·mL^−1^)	Addition content (pg·mL^−1^)	The detection content (pg·mL^−1^, *n* = 5)	RSD (%, n = 5)	Recovery (%, n = 5)
101	50	151, 148, 149, 154, 149	2.4	98.4
101	200	307, 303 305, 305, 310	3.7	102.5
101	500	598,593,602, 593,595	3.4	99

## Conclusion

To sum up, a novel PEC immunosensor was constructed on the basis of SnS_2_@mpg-C_3_N_4_ nanocomposite for detection of PSA. In this study, mpg-C_3_N_4_ with the large specific surface area was used as loader which could provide plenteous chemical sorption sites for the SnS_2_. In addition, mpg-C_3_N_4_ and SnS_2_ formed good energy level matching, which promoted the transfer of electrons and inhibited the recombination of holes and electrons. The PEC immunosensor’s linear detection range was 50 fg·mL^−1^ to 10 ng·mL^−1^, and the detection limit was 21 fg·mL^−1^ (S/N = 3). The PEC immunosensor based on SnS_2_@mpg-C_3_N_4_ nanocomposite has high stability, good selectivity and satisfactory sensitivity, which can provide a detection platform to detect other substances, such as HCG, DNA and cells, *etc*.

## Methods

### Materials and reagents

Tin tetrachloride hydrate (SnCl_4_·5H_2_O) and thioacetamide were obtained from Macklin Biochemical Co., Ltd (Shanghai, China). Dicyandiamide, urea and nitric acid were all purchased from Damao Chemical Reagent Factory (Tianjin, China). NHS and EDC were both purchased from Aladdin Reagent Database Inc. (Shanghai, China). Bovine serum albumin (BSA) was purchased from Sigma-Aldrich (Beijing, China). PSA and anti-PSA were both purchased from Shanghai Linc-Bio Science Co., Ltd., China. All other chemicals were analytical grade and used without further purification. Phosphate buffer solution (PBS) is a mixed solution of disodium hydrogen phosphate (Na_2_HPO_4_·12H_2_O, 0.1 mol·L^−1^) and potassium phosphate monobasic (KH_2_PO_4_, 0.1 mol·L^−1^), which was used for the preparation of the antibody, antigen and washing buffer solution. All aqueous solutions were prepared by using ultrapure water (Milli-Q, Millipore).

### Apparatus

FESEM images were obtained by using a field emission scanning electron microscope (Zeiss, Germany). TEM images were obtained from a JEOL JEM-2100F TEM (Japan). Fourier trans-form infrared spectoscopy was gotten from Bruker VERTEX 70 spectrometer. D8 focus diffractometer (Bruker AXS, Germany) was used to obtain XRD patterns. UV–vis measurements were carried out by using a UV/vis spectrometer (TU-1901). N_2_ adsorption–desorption isotherms were measured at 77 K on a Tristar II 3020 surface area and porosity size analyzer (Micromeritics Instrument Corporation, USA) and the Brunauer–Emmett–Teller (BET) method was used to calculate the surface areas of the samples. The pore size distributions were derived from the desorption branches of the isotherms using the Barrett–Joyner–Halenda (BJH) method. EIS was performed on an autolab potentiostat/galvanostat (Zahner, Germany) with a three electrode system. PEC measurements were all carried out on an electrochemical workstation (Zahner Zennium PP211, Germany). The distance between the light source and the working electrode was 10 cm. All experiments were carried out with a conventional three electrodes system at room temperature by using the ITO as working electrode, a platinum wire as counter electrode and a potassium chloride (KCl) saturated as calomel reference electrode (SCE).

### Synthesis of mpg-C_3_N_4_ and SnS_2_@mpg-C_3_N_4_ nanocomposite

The mpg-C_3_N_4_ was synthesized via pyrolysis of urea and dicyandiamide in air atmosphere according to the previous report^41^. Firstly, 3.5 g urea and 1.5 g dicyandiamide were milled for about 60 min until they became ultrafine powder. After that, the powder was transferred into muffle and heated to 530 °C for 4 h. The yellow products were then washed with deionized water and dried under vacuum 50 °C for 12 h. In order to improve the solubility of mpg-C_3_N_4_ in water, the yellow product was treated with 5 mol·L^−1^ HNO_3_ and refluxed at 125 °C for 24 h^42^. The refluxed product was then centrifuged and washed with ultrapure water for several times, and dried under vacuum at 50 °C for 12 h.

For the synthesis of SnS_2_@mpg-C_3_N_4_ nanocomposite, 13 mL of the volume fraction of 5% acetic acid solution was prepared at first, then 0.061 g of mpg-C_3_N_4_ was added to it and sonicated for 0.5 h, followed by addition of 0.584 g SnCl_4_·5H_2_O and 0.251 g thioacetamide to the above solution during stirring. When the solution was stirred into a homogeneous solution, it was transferred into teflon-sealed autoclave and heated to 180 °C for 12 h to complete the reaction. Then, the resulting product was centrifuged and washed with ultrapure water and ethanol thoroughly in sequence. At last, it was dried under vacuum at 35 °C for 12 h. The resulting faint yellow power was mpg-C_3_N_4_ with the carboxyl groups.

### The construction of label-free PEC immunosensor

Before preparation, the ITO substrates were all cut into 2.5 × 1.0 cm^2^ pieces. After that, the ITO substrates were immersed in 1 mol·L^−1^ NaOH solution overnight, then they were sonicated in acetone, ethanol and distilled water sequentially for about 30 min, and then they were dried with a nitrogen stream for future use^43^.

For fabrication of the immunosensor, firstly, 3 mg of SnS_2_@mpg-C_3_N_4_ powder was dispersed in 1 mL of distilled water, and then 6 μL of the homogeneous suspension was dropped onto a piece of ITO slice. After drying in air, 4 μL of EDC/NHS mixed aqueous solution which contained 1 × 10^−2^ mol·L^−1^ of EDC and 2 × 10^−2^ mol·L^−1^ of NHS was dropped on the modified electrode and until it was naturally dried to wet film state, followed by rinsing off the redundant EDC and NHS with washing buffer. Then, 6 μL anti-PSA solution with a concentration of 10 μg·mL^−1^ was dropped on the above electrode and dried to wet film state at 4 °C, followed by rinsing off physically absorbed anti-PSA. After that, 3 μL of 1% BSA with a mass fraction of 1% was immobilized onto the modified electrode to block non-specific binding sites and dried to wet film state at 4 °C, followed by washing with washing buffer thoroughly. At last, 6 μL of PSA solution with different concentrations were dropped onto different electrodes and incubated at 4 °C, and then washed them with the washing buffer thoroughly. The resulting electrodes were finally employed as a label-free PEC immunosensor for the next PEC tests.

### Photoelectrochemical detection

The experiment of photoelectrochemical detection was implemented in PBS whose pH was 7.0 and the concentration of AA was 0.2 mol·L^−1^. The photocurrent was received from a photoelectrochemical workstation at a bias voltage of 0 V with a light intensity of 180 W·m^−2^ and wavelength of 430 nm at room temperature.

## Electronic supplementary material

Supplementary Information
