# Supplementary material for: Visible-light driven Photoelectrochemical Immunosensor Based on SnS2@mpg-C3N4 for Detection of Prostate Specific Antigen
Source: Sci Rep. 2017 Jul 5;7:4629. doi: 10.1038/s41598-017-04924-x (PMC5498603; doi:10.1038/s41598-017-04924-x)
Supplement: Supplementary file 1 — Supplementary Information [file 41598_2017_4924_MOESM1_ESM.doc]

*Supporting Information*

**Visible-light driven Photoelectrochemical Immunosensor Based on SnS_2_@mpg-C_3_N_4_ for Detection of Prostate Specific Antigen**

Yifeng Zhang^a^, Yixin Liu^a^, Rongxia Li^b^, Malik Saddam Khan^a^, Picheng Gao^b^, Yong Zhang ^a*^, Qin Wei^a^

^a^Key Laboratory of Chemical Sensing & Analysis in Universities of Shandong, School of Chemistry and Chemical Engineering, University of Jinan, Jinan 250022, PR China

^b^Shandong Liyuan Kangsai Environmental Consulting Co. Ltd.

Yifeng Zhang (ifengzhang2014@163.com)

Yixin Liu (E-mail: [hxhglyx@163.com](mailto:hxhglyx@163.com))

Rongxia Li (E-mail: lirx1030@163.com)

Malik Saddam Khan (E-mail: chemistsaddam@qq.com)

Picheng Gao (E-mail:gaopicheng1990@163.com)

Yong Zhang^*^ (E-mail: yongzhang7805@126.com)

Qin Wei (E-mail: sdjndxwq@163.com)

*Corresponding authors. Tel.: ﹢86 531 82767872; Fax: ﹢86 531 82765969.

E-mail addresses: yongzhang7805@126.com (Yong Zhang),


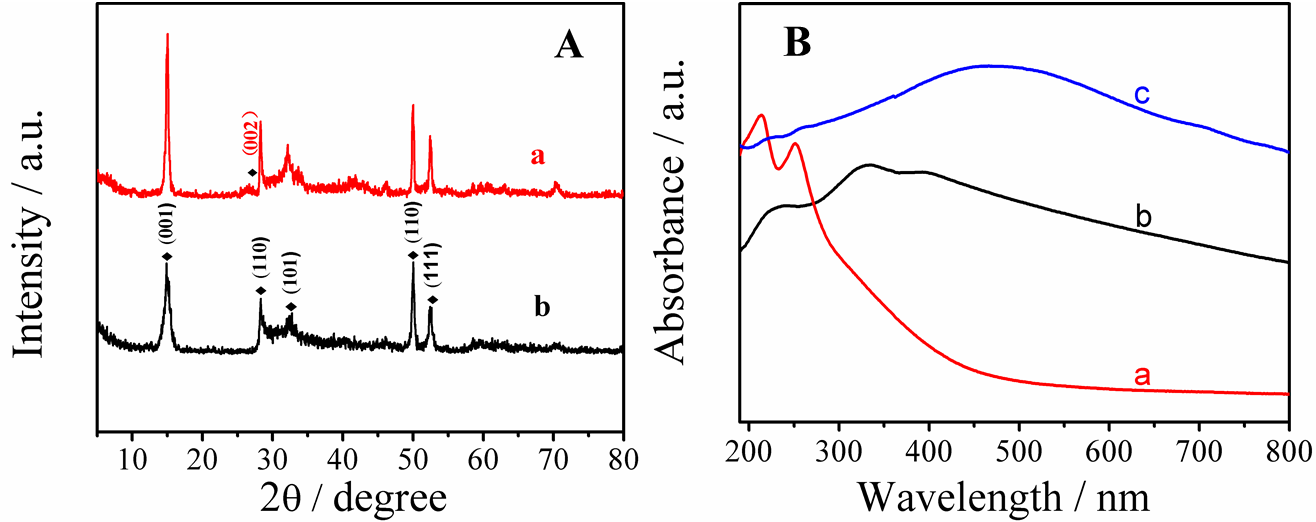


**Fig S1.** (A) XRD patterns of (a) SnS_2_@mpg-C_3_N_4_, (b) SnS_2_. (B)UV-vis absorption spectrum of (a) SnS_2_, (b) mpg-C_3_N_4_, (c) SnS_2_@mpg-C_3_N_4_.


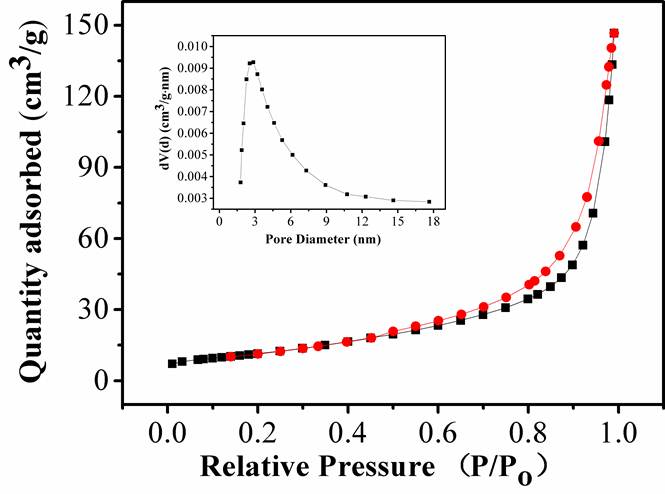


**Fig S2.** Nitrogen adsorption–desorption isotherm and the corresponding BJH pore size distribution for the synthesized mpg-C_3_N_4_.


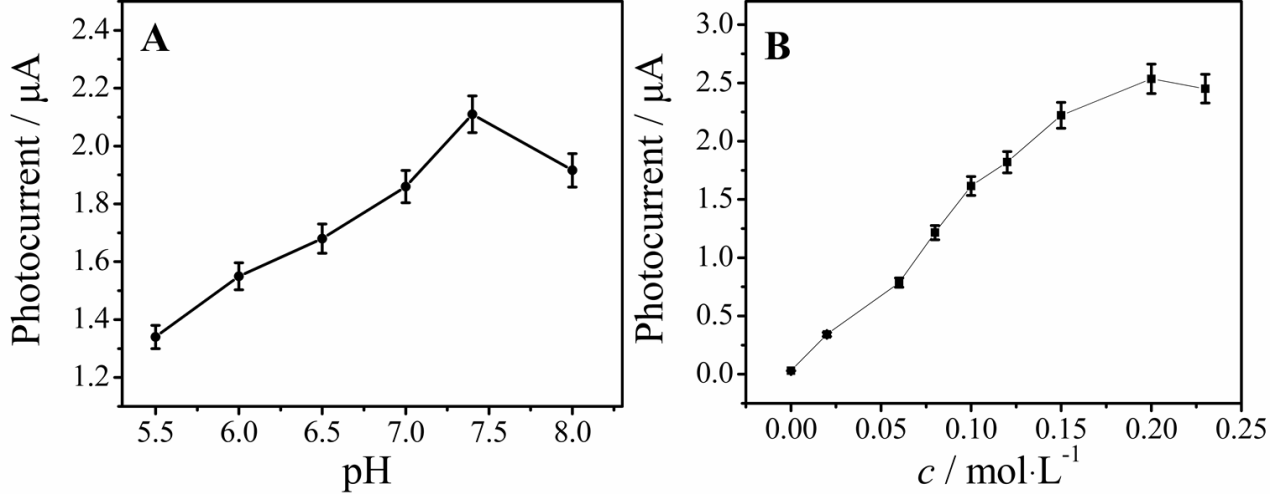


**Fig S3.** Effects of (A) pH and (B) AA concentrations on the photocurrent responses of the PEC immunosensor

**Table S1.** Comparison of different methods for the detection of PSA

| Method | Linear range  (ng·ml^−1^) | Detection Limit  (ng·ml^−1^) | Reference |
| --- | --- | --- | --- |
| Fluorescence Immunoassay | 1.7× 10^0^ ~ 1 × 10^2^ | 1.4 × 10^-1^ | [1](#_ENREF_1) |
| Surface plasmon resonance | 1 × 10^-1^ ~ 5× 10^1^ | 9.1 × 10^-2^ | [2](#_ENREF_2) |
| Electrochemical Immunoassay | 5× 10^-2^ ~ 5 × 10^0^ | 1.3× 10^-2^ | [3](#_ENREF_3) |
| ELISA | 1 × 10^-5^ ~ 1 × 10^-1^ | 4.1 × 10^-6^ | [4](#_ENREF_4) |
| Surface acoustic wave | 1 × 10^1^ ~ 1 × 10^2^ | 1 × 10^1^ | [5](#_ENREF_5) |
| nanomechanical resonators | 5 × 10^-5^ ~ 5× 10^1^ | 5 × 10^-5^ | 6 |
| This work | 5 × 10^-5^ ~ 1 × 10^1^ | 2.1 × 10^-5^ |  |

## References

1 Tajudin, A. A. *et al.* INTEGRATED ACOUSTIC IMMUNOAFFINITY-CAPTURE (IAI) PLATFORM FOR DETECTION OF PSA FROM WHOLE BLOOD SAMPLES. *Lab on A Chip* **13**, 1790-1796 (2013).

2 Ertürk, G., Özen, H., Tümer, M. A., Mattiasson, B. & Denizli, A. Microcontact Imprinting Based Surface Plasmon Resonance (SPR) Biosensor for Real-time and Ultrasensitive Detection of Prostate Specific Antigen (PSA) From Clinical Samples. *Sensors & Actuators B Chemical* **224,** 823-832 (2015).

3 Mao, K. *et al.* Label-free electrochemical immunosensor based on graphene/methylene blue nanocomposite. *Analytical Biochemistry* **422**, 22-27 (2012).

4 Liang, J. *et al.* Silver nanoprism etching-based plasmonic ELISA for the high sensitive detection of prostate-specific antigen. *Biosensors & Bioelectronics* **69**, 128-134 (2015).

5 Zhang, F. *et al.* A Microfluidic Love-Wave Biosensing Device for PSA Detection Based on an Aptamer Beacon Probe. *Sensors* **15**, 13839-13850 (2015).

6 Waggoner, P. S., Varshney, M. & Craighead, H. G. Detection of prostate specific antigen with nanomechanical resonators*. Lab on a Ch*i**p** 9, 3095-3099 (2009).
